# A Rapid and Simple Method for Purification of Nucleic Acids on Porous Membranes: Simulation vs. Experiment

**DOI:** 10.3390/mi13122238

**Published:** 2022-12-16

**Authors:** Angela Fonseca-Benitez, Consuelo Romero-Sánchez, Sandra Janneth Perdomo Lara

**Affiliations:** 1 Cell and Molecular Immunology Group, El Bosque University INMUBO1, Bogota 11001, Colombia; 2 School of Dentistry, Universidad El Bosque, Bogota 11001, Colombia

**Keywords:** lab on a chip, microfluidic interface, clinical diagnosis, saliva

## Abstract

Paper-based microfluidic systems have emerged as one of the most promising technologies for developing point-of-care diagnostic platforms (POCT) for detecting and monitoring various diseases. Saliva is a non-invasive biofluid easily collected, transported, and stored. Due to its accessibility and connection to systemic diseases, saliva is one of the best candidates for medical advancement at the point of care, where people can easily monitor their health. However, saliva is a complex mixture of DNA, RNA, proteins, exosomes, and electrolytes. Thus, nucleic acid separation from the salivary components is essential for PCR applications. Paper membranes are a highly porous and foldable structure capable of transporting fluids without pumps and sophisticated systems. The current work presents an insight into simulations for nucleic acid extraction on three types of porous paper membranes for use in point-of-care devices. The flow fluid model is solved on a COMSOL Multiphysics 5.3 free version platform, and the results are compared with experimental assays. The results show that pore uniformity, wet strength, porosity, and functional groups of MF1™ and Fusion 5™ paper membranes are vital parameters affecting nucleic acid extraction and PCR amplification efficiency.

## 1. Introduction

The necessity to quickly identify nucleic acids for disease diagnostic in low- and middle-income countries has led to the development of low-cost POCT systems. The POCT promises to perform diagnostic tests outside the laboratory with rapid and reliable results [1]. In diagnostic applications, nucleic acid-based analysis has many advantages such as sensitivity, specificity, rapid results, and flexibility [2]. However, carrying out nucleic acid amplification and detection is not an easy task. Concerning this, developing a rapid method, equipment-free, and straightforward nucleic acid extraction is a precise process, which is traditionally time-consuming and requires trained technicians and multiple steps. [3]. Thus, to realize a POCT diagnostic tool, the extraction, amplification, and detection steps should be integrated [4].

Paper membranes emerged while searching for a low-cost, portable, biocompatible, and easy-to-modify material for fabricating diagnostic devices [5]. These membranes have exhibited good nucleic acid extraction and detection at POCT. Moreover, these have easy use and storage. However, it is necessary to choose the paper adequately based on its physical and chemical properties, considering the specific POCT applications.

Similarly, liquid biopsy allows for the detection, analysis, and identification of different diseases in body effluents such as blood, urine, sputum, bronchial fluid, and saliva. This latter element has many advantages of being used as a biofluid. As it represents a non-invasive procedure, its collection is fast, inexpensive, and too easy. The saliva is called “the mirror of the body” because it reflects the health–disease general state of the body. Saliva allows the identification of DNA, RNA, proteins, exosomes, electrolytes, etc. [6]. However, saliva use, like liquid biopsy in clinics and POCT devices, requires more standardization in collection and evaluation procedures.

Consequently, this study compared the performance of three common paper-based materials for nucleic acid extraction from saliva samples. We performed flow fluids simulations with COMSOL Multiphysics software. Additionally, saliva as a model sample was used to evaluate the efficiency of these materials’ nucleic acid extractions in experimental assays.

## 2. Materials and Methods

### 2.1. Saliva Rheology Measurement

Rheology was measured because saliva shows high viscosity, elasticity, and adhesiveness features that can affect fluid displacement through membranes. Thus, unstimulated and stimulated saliva samples were analyzed using a Modular Compact Rheometer (MCR) series, MCR 92. Each sample (Saline solution, saliva stimulated, and saliva unstimulated) was loaded between 3 mm diameter plates set at an initial gap of 2 mm, requiring 100 µL of the sample. The capillary formation was set to a sample end height of 10.8 mm with a stretch time of 50 ms. A total of 21 points were made, 1 point per minute. Surface tension was set to 52 mN m^−1^ based on the literature values. Sample density was set to 1 g cm^−3^. The rheometer plates were cleaned with ethanol, followed by distilled water, and then air-dried between samples.

### 2.2. Simulation Method

In order to obtain an approximation, the present study first simulated saliva flow through porous membranes in the nucleic acid extraction using a free version of COMSOL Multiphysics software Porous Media Flow Module. We selected the three most common Whatman membranes used in lateral flow assays; cotton linter (CF4^®^), glass-bound fiber (MF1^®^), and Fusion 5^®^, a proprietary mix of glass fiber and polymer. The selection of paper substrate depended on various characteristics such as capillary flow time (the time required for a liquid sample to flow through the pores in the lateral direction), the thickness of the paper, pore size, porosity (% of air present in porous structure), and surface quality (Table 1).

### 2.3. Mathematical Modeling

The Lucas–Washburn (L–W) equation was first used for the mathematical model. This equation assumes that fluids are non-Newtonians and that capillary channels all have the same radii. Saliva displacement depends on porous systems’ microstructure, interface tension, fluid property, and fluid−solid interactions. When saliva is in contact with the paper, the interactions between the solid–liquid interfaces spread the saliva into the porous substrate, called the wet-out process. Finally, the fluid front is wicking along with the dry porous media in its movement. The equation is as follows:

(1) $$l(t)=2\cdot \sqrt{\frac{K\gamma cos\theta }{\varphi \mu r\alpha }}\cdot \sqrt{t}$$

where *l*(*t*) represents the fluid length traveled in the paper after the time (*t*), and *k* is permeability, understood as a measure of flow that occurs through a substrate. This process depends upon pore size and geometry; *µ* is the fluid viscosity, and *t* is the liquid penetration time.

Moreover, Darcy’s law was also used to simulate the saliva flow behavior through a porous membrane using the distribution of the porous media properties (porosity, permeability, and saturation), in which the pressure gradient is the major driving force, and the porosity of membranes is very small. The equation describes a linear relationship between the velocity field *µ* (m/s) and the gradient of the pressure *p* (Pa):

(2) $$q=-\frac{k}{\mu }\;\nabla p\;$$

where *q* = instantaneous flow rate, *k* = permeability, and *µ* = the dynamic viscosity of the fluid and = pressure drop, respectively. This work considers a rectangular porous paper strip length *L*, uniform width w, and thickness h. *ra* is the average pore radius of the paper. When a small amount of liquid is dropped onto the paper substrate, three types of interfacial layers are formed due to surface tension, i.e., solid–liquid interface, liquid–vapor interface, and vapor–solid interface. The fluid movement is influenced mainly by the physical and geometrical properties of porous media, such as the physical and rheological properties of saliva. Thus, COMSOL Multiphysics software couples the Multiphase Flow in Porous Media, with Darcy’s law and Phase Transport in Porous Media interfaces. The Phase Transport in Porous Media interface follows separate equations for the volume fraction (*Si*), these depend on whether there is a wetting or no wetting fluid *i*:

(3) $$\frac{\partial }{\partial t}\left(\varepsilon_{p\;}\rho_{i}s_{i}\right)+\nabla .\left(-\rho ik\frac{k_{ri}}{\mu_{i}}\left(\nabla pi-pig\right)\right)=Q_{i}=0$$

where *ε_p_* is the porosity, *κ* is the permeability (m^2^), *κ_ri_* is the relative permeability (a function of saturation for a given fluid), *μ_i_* is the fluid’s dynamic viscosity (Pa·s), *p_i_* is the pressure (Pa), and *ρ_i_* is the fluid density (kg/m^3^) of phase *i*.

### 2.4. Experimental Method

#### 2.4.1. Paper Membranes

As described above, the three Whatman^®^ qualitative membranes used were e: CF4 (100% cotton linter), MF1^™^ paper (bound glass fiber), and Fusion 5^™^ (patent membrane) and were purchased from Cytiva. Each paper was cut into 0.5 × 6 cm strips. The physics and geometric characteristics are described above (Table 1).

#### 2.4.2. Sample and Paper Preparation

The unstimulated (US) and stimulated saliva (SS) samples used in this work were collected into sterile 15 mL tubes from healthy volunteers. These samples were used immediately (as fresh samples). It was mixed with blue food color (1:1) to facilitate the displacement observation. Additionally, a 10 µL lysis buffer was deposited onto the strips using a micropipette. Then, the lysis buffer was dried on the paper; each membrane strip was placed on a horizontal surface.

#### 2.4.3. Paper-Based Nucleic Acid and Protein Extraction and Quantification

**Figure 1:**
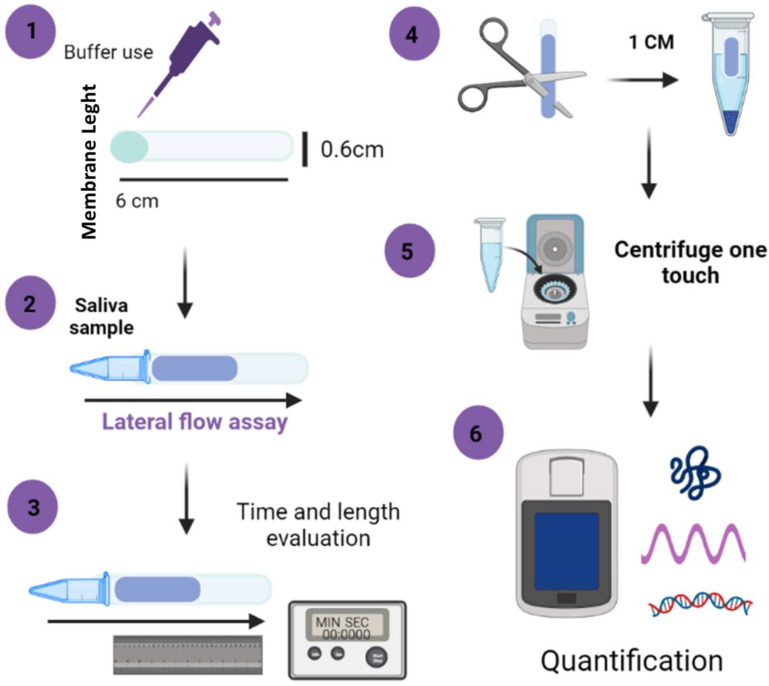
Schematic representation of paper-based nucleic acid extraction assay: steps 1–3, description of lateral flow assay; steps 4–6, process of DNA, RNA, and protein quantification using Qubit Fluorometer.

This system consisted of a porous membrane in contact with a 100 µL saliva sample in 0.5 mL RNA/DNA-free tubes used as a reservoir. The flow distance using a conventional rule and timer was measured. The wicking into the membrane occurred via capillary action for times t > 0 (20, 40, 60, 120, 180, and 240 s), and the liquid–air interface within the porous membrane migrates toward dry regions. The process is described in Figure 1, steps 1 to 3. Subsequently, for the evaluation of nucleic acid and protein extraction from saliva, 1 cm of each membrane at different times was cut and transferred to 0.5 mL tubes for the elution step. For this, 100 µL of grade 1 water was added, and the membranes were centrifuged at 1000 rpm for 1 min. Then, DNA, RNA, and protein concentrations were measured using a Qubit 4 fluorometer (Thermo Fisher Scientific, US), following the manufacturer’s instructions to measure nucleic acid (DNA/RNA) and protein accurately. Briefly, 2 µL per sample was mixed with 199 µL working solution and 1 µL of the fluorochrome (Figure 1, steps 4–6).

#### 2.4.4. Efficiency Evaluation of Paper-Based RNA Extraction

Real-time PCR (RT-PCR) was used to verify the success of MF1 and Fusion 5^™^ membranes for RNA extraction from saliva samples. B-Actin housekeeping primer was used as quality control of RNA obtained (sense ATTGCCGACAGGATGCAGA and antisense ATTGCCGACAGGATGCAGA). The elute RNA from 0.6 × 6 cm membranes MF1 and Fusion 5^™^ were evaluated for amplification. The PCR mix contained 10 μL of the amplification reagents, comprising 0.2 μL forward and reverse primers, 5 μL of Luna® Universal One-Step RT-qPCR (New England Biolabs NEB, UK), 0.5 μL of retrotranscriptase enzyme, 2.3 μL of ddH2O, and 2 μL of RNA template solution. CT values were evaluated with RT-PCR.

## 3. Results

### 3.1. Saliva Viscosity

**Figure 2:**
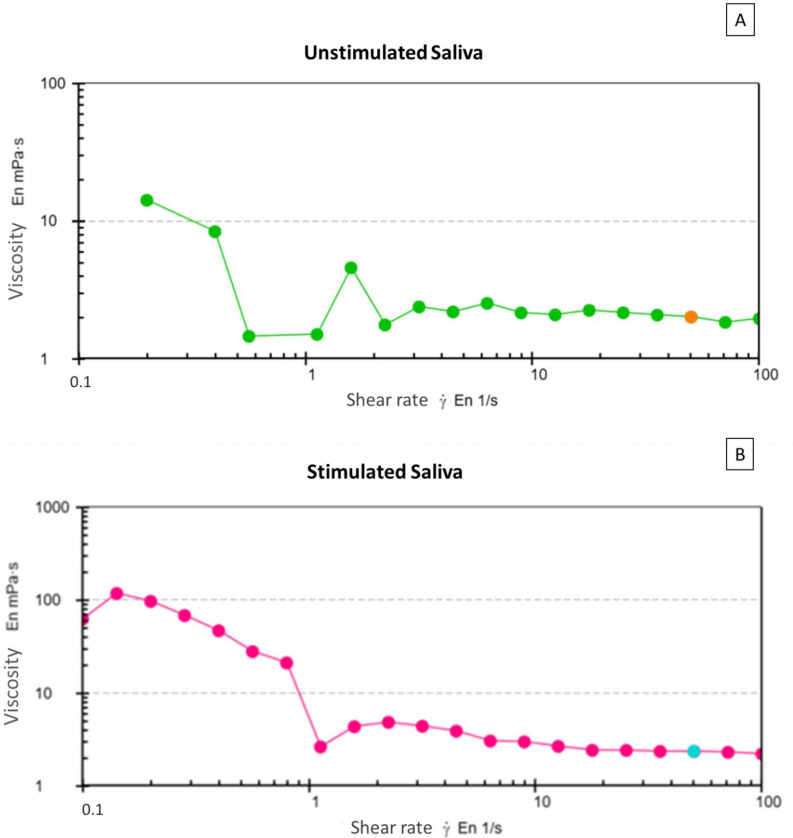
Saliva viscosity: (**A**) unstimulated (green lines) and (**B**) stimulated (pink lines). Saliva viscosity was analyzed using a modular compact rheometer. A total of 21 points were made, 1 point per minute, per each saliva type.

In order to model the saliva flow as a non-Newtonian fluid, we first characterized its viscosity because it can affect the saliva flow for nucleic acid and protein extraction in porous membranes. In this study, we observed that SS had less viscosity (2 mPas), compared with US (2.34 mPas) (Figure 2), suggesting that SS could be more efficient in fluid l flow for POCT applications. Considering the viscosity of SS, these samples were used for the simulation and experimental assays of fluid flow in porous membranes. The unstimulated saliva was not used in the subsequent assays.

### 3.2. Wicking of Saliva in a Paper Strip

Wicking is the phenomenon that occurs when dry porous material is put into contact with a fluid that will absorb it due to capillary forces [7]. The mathematical model represents a paper strip 2D model with a rectangular geometry and a height of 6 cm and width of 0.6 cm. Features of each membrane are described according to the manufacturer’s specifications (Table 1). In addition, the model solves for air saturation and saliva pressure using the equations previously described. The different material parameters used in this model are presented in Table 2. The paper strip was filled with air with an initial saliva saturation of 0.01, a viscosity of 0.002 Pa·s, and a density of 1000 kg/m³.

**Figure 3:**
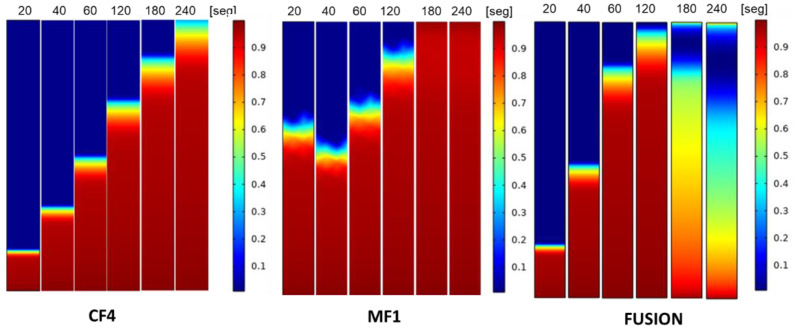
Saliva flow simulation results. Saliva saturation in the papers strips after 20, 40, 60, 120, 180, and 240 s, simulation results are shown.

The saliva flow on membranes was measured for 20, 40, 60, 120, 180, and 240 s. The simulation results showed saturation at 240 s, related to saliva properties and membranes (CF4™, Fusion 5™, and MF1™). After 120 s, the paper strips were already soaked with saliva as a function of time. Figure 3 shows the saliva saturation at different times. The behavioral details of saliva flow were subsequently determined through comparison with the experimental tests.

### 3.3. Lateral Flow Assay

**Figure 4:**
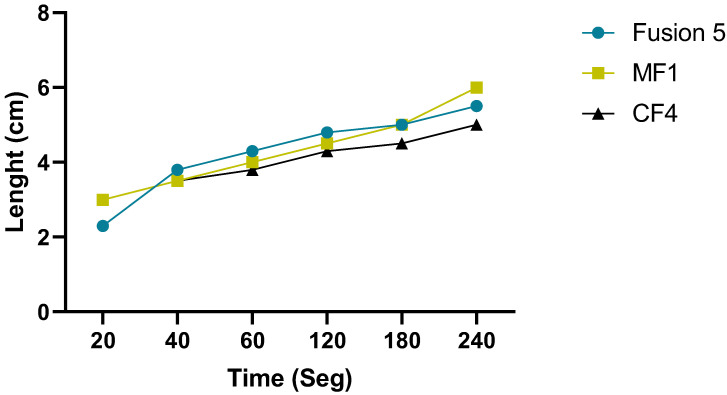
The rate of movement of saliva through a porous membrane. Lateral flow of saliva in membranes (Fusion 5™, MF1™, and CF4™) with a rectangular geometry of 6 cm in length and 0.6 cm in width at 20, 40, 60, 120, 180, and 240 s.

The results obtained using simulation in the current work were compared with experimental data to verify the mathematical model accuracy. The lateral flow of saliva through porous membranes was similar for each paper (Figure 4). The measurement of the saliva flow began at 20 s with a path of 2 cm. The saliva movement rate through a porous membrane is expressed as the time taken for the liquid to move a certain distance. Results showed that the saliva moved between 4 and 5 cm in length and times at 60 and 180 s in the three membranes, reaching 6 cm at 240 s for Fusion 5™ and MF1. However, in the CF4 paper, saliva moved 5 cm, thus not achieving membrane length, assuming its saturation (Figure 4).

### 3.4. Comparison of Saliva Flow through Porous Membranes between Simulation and Experiment

#### 3.4.1. Absorption Saliva in Each Membrane

**Figure 5:**
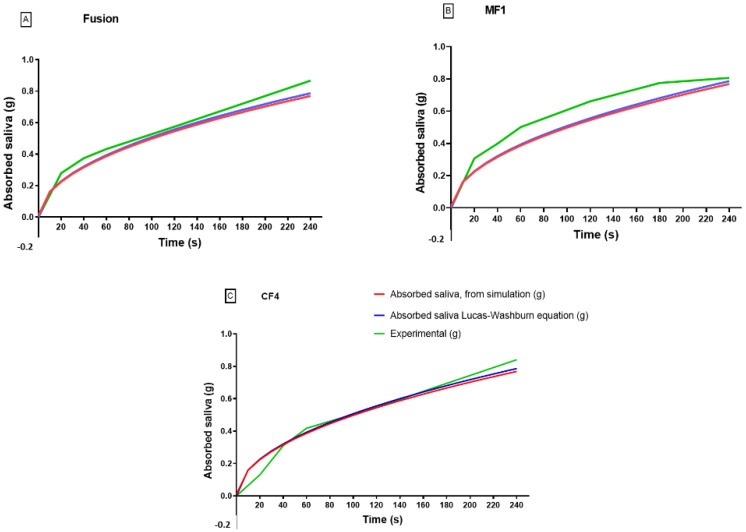
Comparison of the simulation and numerical experimental data is shown for capillary absorption of saliva in paper strips (time vs. absorbed saliva): (**A**) in a 6 cm paper strip of CF4™ membrane; (**B**) Fusion 5™ membrane; (**C**) MF1™ membrane.

The saliva absorption in a paper strip at a 6 cm height employing the L–W equation, numerical simulation, and experimental results are shown in Figure 5. The model used capillary radius (Rc) as half of the pore diameter for each membrane. These data are given by the manufacturer (Whatman). The experimental and simulated data showed similar results. In the case of the Fusion 5™ membrane, saliva absorption reached 0.7 and 0.8 g in the simulation and experimental results, respectively. The behavior of the three curves was similar in Fusion 5™ and CF4™ membranes.

In contrast, the experimental curve (green) in the MF1 membrane achieved higher saturation than the simulation results (red). Nevertheless, all assays obtained 0.8 g of absorption at 240 s. The data from the L–W model were not comparable with the experimental results because, in the L–W equation, only the capillary height is considered to vary with time, not the retardation in saliva flow velocity or viscosity resistance. Thus, all of the properties of the saliva would be considered. There is a possibility that pores/paper strips were blocked due to the solid content and because saliva is a non-Newtonian fluid with viscosity that decreased with increasing shear and velocity rates (Figure 5).

**Figure 6:**
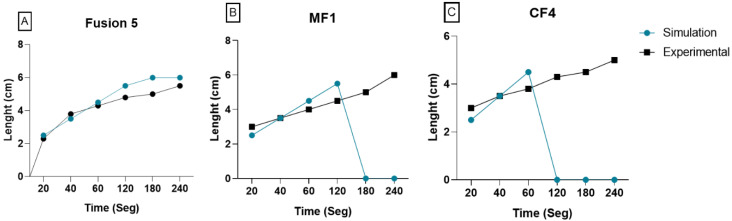
Comparison of lateral flow simulation and experimental. Comparison of LFA of saliva in each paper. Distance versus time was measured in both simulation and experimental models: (**A**) Fusion 5™ membrane; (**B**) MF1™; (**C**) CF4™.

Additionally, the wicking in a 6 cm height paper strip was compared in the time vs. length function of both simulated and experimental results. Mostly, the results were comparable. Fusion 5™ membrane showed the same behavior during the first 60 s with a flow of approximately 4.2 cm in 40 s. However, subsequent changes oscillated between 0.1 and 0.5 cm. On the other hand, CF4 and MF1 membranes kept a constant flow rate until 60 and 120 s, respectively. Similarly, saliva toured 6 cm at 240 s, while simulation results showed that saliva reached 6 cm at 120 s, suggesting possible membrane saturation (Figure 6).

**Figure 7:**
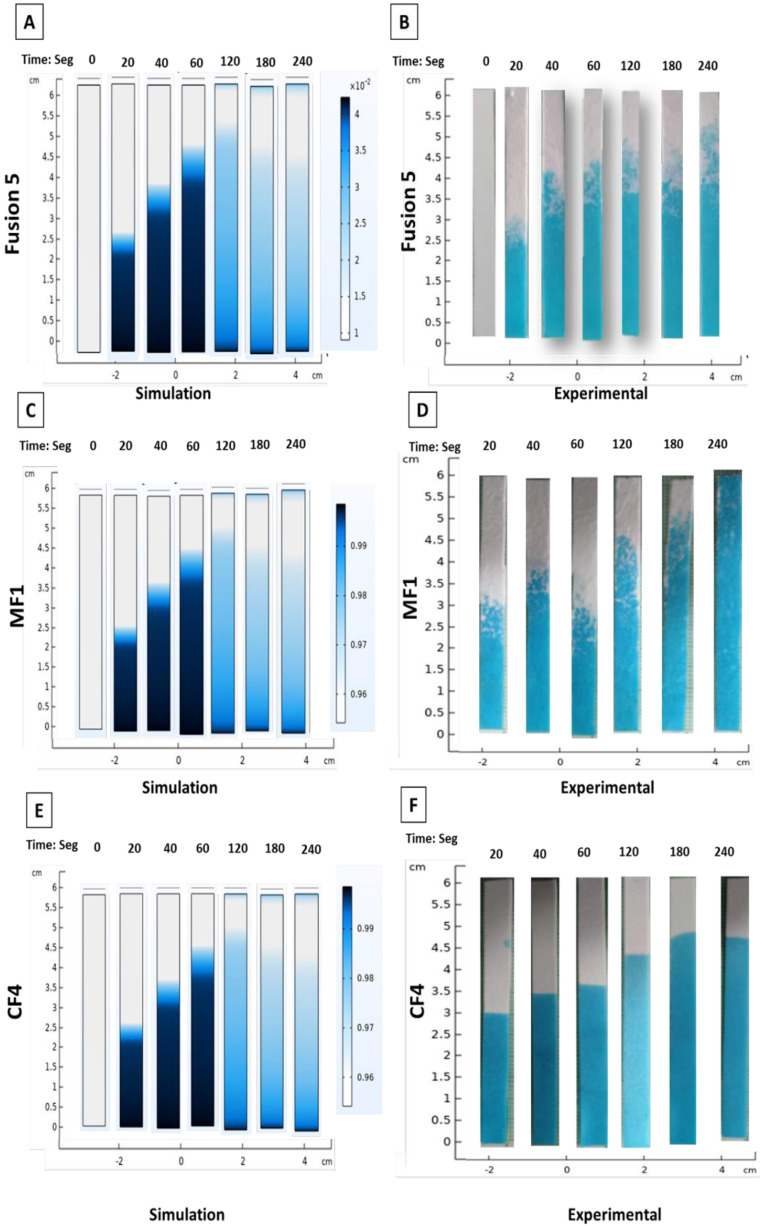
Saliva fraction in simulation vs. experiment. Simulation (**A**,**C**,**E**) and experimental (**B**,**D**,**F**) saliva saturation results are shown per membrane. The saliva chemistry features were introduced to COMSOL software for saturation of each membrane evaluation. The saliva was mixed with blue food coloring to better follow the saliva flow.

In the same way, it was observed that saliva saturation in Fusion 5™ and CF4™ membranes reached the lengths of 2, 3, and 4 cm at 20, 40, and 60 s, respectively, both simulations were experimental with a running front of 0.5 cm. After that, the flow was stopped, and saturation was achieved at 5 cm for 180 s. During the first 60 s, MF1™ showed similar behavior to that of other membranes. However, saturation was reached at 240 s, completing the total length membrane (Figure 7). These results demonstrated the superior sample wicking rate of the MF1™ (fiberglass) and Fusion 5™ material (a polymer/glass fiber matrix) over CF4™ membranes.

#### 3.4.2. Acid Nucleic and Protein Quantification between Stimulated (SS) and Unstimulated Saliva (US)

Solutions for rapid nucleic acid purification are necessary for POCT applications. In this study, the performance of three membranes for nucleic acid extraction was evaluated.

#### 3.4.3. DNA Quantification Per Length and Time

**Figure 8:**
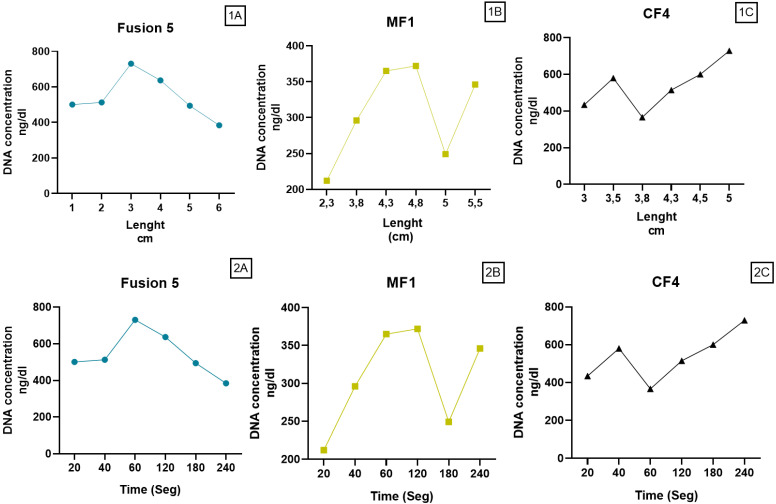
DNA concentration per length (1) and time (2). Comparison of DNA efficiency extraction per membrane between length (1**A**–1**C**), and time (2**A**–2**C**). Distance versus time was measured in experimental models.

DNA concentration per membrane length and saliva flow time was quantified (2A–2C). Results showed that concentration was 400 ng/µL during the first 2 cm for Fusion 5 and CF4; however, MF1 was lower (200 ng/µL) until 4 cm, and it did not reach 400 ng/µL. The maximum concentration achieved was 700 ng/µL using CF4™ membrane at 5 cm in length. It can be observed in Figure 8**.**

**Figure 9:**
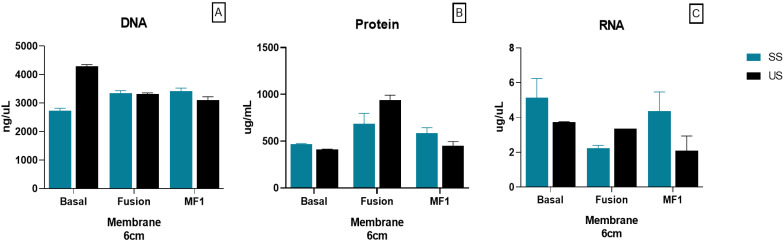
Nucleic acid and protein concentration in SS and US saliva differences after extraction using membranes: (**A**) DNA, (**B**) protein, and (**C**) RNA.

Ideal paper-based material for POCT application should have characteristics such as the high adsorption of nucleic acid and low adsorption of non-specific substances. Given the low DNA quality obtained using the CF4™ membrane, we decided only to evaluate RNA, DNA, and protein extraction to Fusion 5™ and MF1™, comparing stimulated (SS) and unstimulated (US) saliva. Additionally, the basal concentration of each saliva type was measured. Fusion 5™ and MF1™ did not show differences in DNA concentration between SS and US, nor one with the other. However, concerning protein, better results were obtained using MF1™. Similarly, the concentration did not increase significantly. Lastly, RNA extraction was higher with MF1™, showing differences linking SS and US, with better results for SS (Figure 9).

#### 3.4.4. Efficiency Evaluation of Membranes for Nucleic Acid Extraction

**Figure 10:**
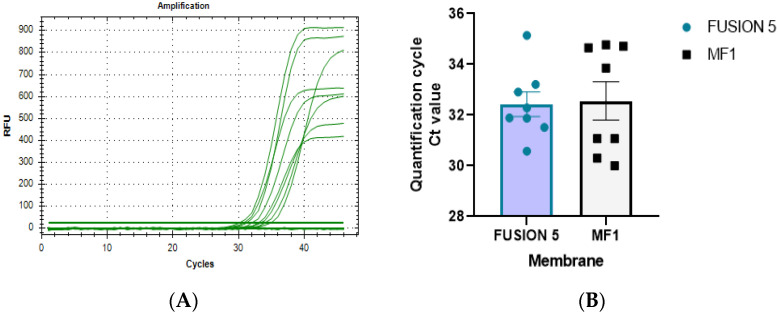
The quantitative results of Fusion 5 and MF1 nucleic acid extraction materials: (**A**) amplification curve; (**B**) distribution Ct value.

Based on the above RNA concentration of Fusion5 and MF1, their membrane performances were also evaluated via RT-PCR (quantitative detection). In actual clinical application, the whole saliva is an emergent-used clinical sample. Thus, as a model, stimulated saliva was used to verify the performance of membranes in this study. The results illustrated that the extraction performances of Fusion and MF1 were similar. This demonstrated that both membranes could be used for nucleic acid extraction. Moreover, they showed an excellent, cleaner capacity to purify impurities in the RNA/DNA solution (Figure 10).

## 4. Discussion

Liquid biopsy is a non-invasive alternative sample for diagnosis (e.g., blood, urine, saliva). However, a complex mixture of analytes in each sample makes them difficult to be directly analyzed [8]. Specifically, saliva is a highly viable biofluid for diagnostic application because it includes various components such as DNA, RNA, proteins, metabolites, and microbiota. Moreover, its collection is non-invasive, straightforward, easily accessible, and stored [9]. Therefore, its pretreatment is vital to nucleic acid extraction, and the standardization of the protocols for removing any components that can interfere with PCR results and retrieving only the target analyte for downstream analysis is essential for its use in POCT devices.

With the advances in diagnostic devices, it is crucial to develop paper-based nucleic acid extraction methods [10]. Hence, in the present study, we compared three Whatman membranes using lateral flow devices for POCT applications. Capable devices offer the same sensitivity and specificity as molecular diagnostic methods. However, several challenges in processing saliva are well-known since it is a non-Newtonian fluid with variations in sample viscosity and composition between stimulated and unstimulated saliva [11]. Although many substrates can capture nucleic acids, low-cost membranes can rapidly purify nucleic acids from crude saliva samples and elute them directly into the amplification mix or lateral flow assay without using micropipettes.

The method described in this study takes advantage of the membrane pore size properties, SS saliva viscosity, and differences between nucleic acids and other compounds concerning their capture and retention kinetics by membranes. Additionally, the saliva flow process through porous membranes is essential to purify nucleic acids. The goal of the fluid flowing through the membrane is to remove interferents, producing a solution containing a DNA/RNA target [12]. Here, we first simulated the wicking phenomenon of the saliva in porous membranes and observed the capillary flow in paper strips of three materials. Due to wettability, the porous surface wets and helps the saliva wick through the porous membranes. The wicking of saliva occurs spontaneously in porous media, without extra energy due to the inherent capillary action [13].

Although paper membranes consist of randomly deposited materials with different porosities, the L–W model considers paper bundles of interconnected capillary tubes. However, a deviation with experimental results was observed because the paper strips used (CF4, Fusion5, and MF1) did not consist of bundles of capillary tubes [14]. MF1™ and Fusion 5™ exhibited asymmetric porous structures, whereas that of CF4™ was symmetrical; this porosity distribution is directly related to the particle retention capabilities of the paper and flow velocity. One exciting approach was combining porous media properties with the L–W model, considering Darcy’s law, where fluid transport occurs along with the wetted porous media [15]. Different flow speeds can be used in LFAs devices, depending on the requirements for sample volume, assay time, specificity, and sensitivity [16]. In porous membranes, capturing sizeable nucleic acid molecules depends on pore size (Figure 8), whereas other, more minor compounds are rapidly released with fluid movement through the paper. Furthermore, the volume absorbed by membranes, the amount of nucleic acid available in saliva samples for capture, and the interaction with paper substrates influence the extraction.

In addition, surface chemistry properties affect fluid behavior in membranes and can help the adsorption capacity of nucleic acid. In this sense, it was observed that paper-based glass fiber composed of SiO2 (MF1 and Fusion 5) could capture NA negatively charged by positively charged silica [16]. Previously, the wet strength of glass-fiber-based materials has been described as higher than that of cotton-based materials [17].

The results illustrated that the MF1 and Fusion 5 membranes’ extraction performance was highest, compared with that of other paper-based materials. In contrast, the CF4 membrane could not extract DNA from the whole saliva. The all-around performance (i.e., cost, time, operation step, extraction efficiency, and sample volume) of silica membrane was highest compared with that of other paper-based materials described by other authors for extracting nucleic acid from whole saliva [18].

Emerging “point-of-care testing” (POCT) has played an essential role in early disease detection, diagnosis, and maintenance. These have been widely accepted as economical and alternative diagnostic tool tests, especially when access is difficult. An essential feature of paper-based POCT devices is that they must be rapid and robust [19]. However, there are several challenges to be solved to develop future POCT diagnostic devices, including (1) improving speeding nucleic acid extraction and purification within minutes and (2) better POCT methods for extracting nucleic acid for biological fluids [20].

Ideal paper-based materials for POCT applications should have characteristics that allow easy use. These include high efficiency in purifying nucleic acids from samples and the low adsorption of non-specific substances [21]. In relation to this topic, through comparing membrane features, we concluded that Fusion 5 and MF1 had better results, given that DNA concentration was not too high, compared with CF4 results. Knowing the concentration is essential because it can inhibit PCR and obtain false-negative results.

## 5. Conclusions

Our study revealed that 6 cm of paper could purify nucleic acids away from inhibitors. A significant advantage of the method presented here is that the amount of nucleic acid transferred to the amplification reaction would be similar between samples of the same type because the size of the DNA binding surface remained constant.

The theoretical simulation of fluid flow in porous media and experimental validation can significantly contribute to the development of paper-based diagnostic devices to study various diseases, both infectious and tumors.

## Figures and Tables

**Table 1:** Membrane characteristics.

Membrane	Material	Thickness (µm @ 53 kPA)	Wicking Rate (s/4cm)	Pore Size (µm)	Porosity %
Whatman TM CF4	100% cotton linter	482	67.3	11	80
Whatman TM MF1	Bound glass fiber	367	29.7	0.45	69
Whatman TM Fusion 5	Based on proprietary FUSION (single layer matrix Technology)	370	43.9	21 to 181	80

**Table 2:** Parameters used to define the simulation of saliva flow on paper membrane for nucleic acid extraction and separation. The characteristics of the CF4 membrane were taken as an example.

Name	Expression	Value	Description
D2	1 × 10^−5^ [m^2/s]	1 × 10^−5^ m²/s	Diffusion coefficient
epsilon1	0.383	0.383	Porosity
D1	2.15 × 10^−6^ [m^2/s]	2.15 × 10^−6^ m²/s	Diffusion coefficient, 1D
L0	6 [cm]	0.06 m	Paper strip height
W0	L0/8	0.0075 m	Paper strip width
th	0.482 [mm]	4.82 × 10^−4^ m	Paper strip thickness
sigma	72 [mN/m]	0.072 N/m	Surface tension
Rc	2.25 × 10^−6^ [m]	2.25 × 10^−6^ m	Pore radius
pec	2*sigma*cos(theta)/Rc	47,498 N/m²	Entry capillary pressure
lp	2	2	Pore size distribution index
por	0.8	0.8	Porosity
rho_air	1 [kg/m^3]	1 kg/m³	Air density
rho_saliva	1 × 10^3^ [kg/m^3]	1000 kg/m³	Saliva density
mu_air	1.76 × 10^−5^ [Pa*s]	1.76 × 10^−5^ Pa·s	Air viscosity
mu_saliva	0.002 [Pa*s]	0.002 Pa·s	Saliva viscosity

(*) Refers to multiplications.

## Data Availability

Data details about the simulation are shown in the supplementary material.
